# Clinical-histopathological correlation in a case of Coats' disease

**DOI:** 10.1186/1746-1596-1-24

**Published:** 2006-08-30

**Authors:** Bruno F Fernandes, Alexandre N Odashiro, Shawn Maloney, Moyses E Zajdenweber, Andressa G Lopes, Miguel N Burnier

**Affiliations:** 1 Department of Ophthalmology and Pathology. The McGill University Health Center & Henry C. Witelson Ocular Pathology Laboratory. Montreal, Canada; 2 Department of Ophthalmology. Federal University of Sao Paulo – UNIFESP/EPM. São Paulo, Brazil; 3 Department of Ophthalmology. Hospital dos Servidores dos Estado. Rio de Janeiro, Brazil

## Abstract

**Background:**

Coats' disease is a non-hereditary ocular disease, with no systemic manifestation, first described by Coats in 1908. It occurs more commonly in children and has a clear male predominance. Most patients present clinically with unilateral decreased vision, strabismus or leukocoria. The most important differential diagnosis is unilateral retinoblastoma, which occurs in the same age group and has some overlapping clinical manifestations.

**Case presentation:**

A 4 year-old girl presented with a blind and painful right eye. Ocular examination revealed neovascular glaucoma, cataract and posterior synechiae. Although viewing of the fundus was impossible, computed tomography disclosed total exsudative retinal detachment in the affected eye. The eye was enucleated and subsequent histopathological evaluation confirmed the diagnosis of Coats' disease.

**Conclusion:**

General pathologists usually do not have the opportunity to receive and study specimens from patients with Coats' disease. Coats' disease is one of the most important differential diagnoses of retinoblastoma. Therefore, It is crucial for the pathologist to be familiar with the histopathological features of the former, and distinguish it from the latter.

## Background

Coats' disease is a non-hereditary ocular disease, with no systemic manifestation, first described by Coats in 1908[1]. It occurs more commonly in children and has a clear male predominance (69%) [2]. Most patients present clinically with unilateral decreased vision, strabismus or leukocoria [3]. The most important differential diagnosis is unilateral retinoblastoma, which occurs in the same age group and has some overlapping clinical manifestations [4].

Although there are several articles discussing the clinical variations and treatment modalities for Coats' disease, histopathological reports are not seen that often since currently there are conservative ways to treat the disease. Consequently, an enucleation specimen that permits a histopathological study with reference to the basis of the disease is rare.

In this study, we report a case of Coats' disease in a young girl, and evaluate the histopathological abnormalities underlying clinical findings.

## Case report

**Figure 1:**
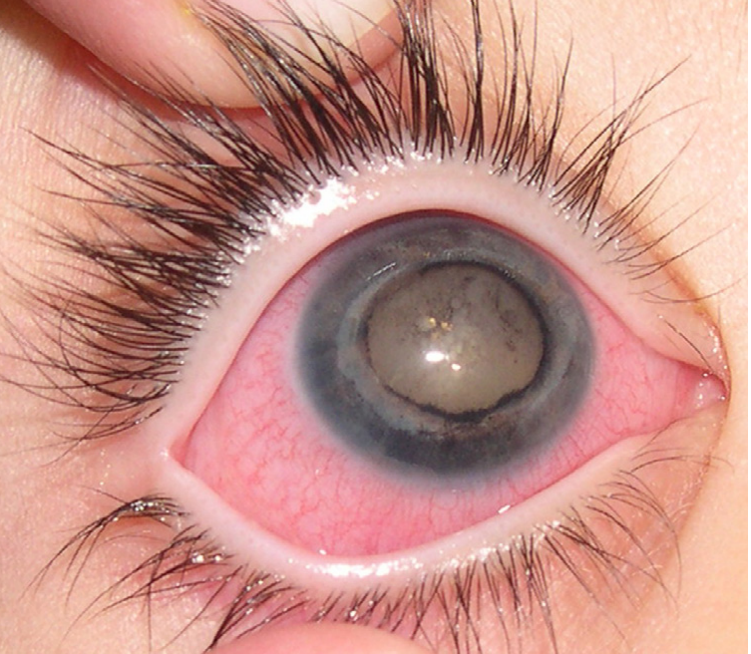
Clinical photography. Conjunctival hyperemia, mild corneal edema, posterior synechiae and cataract.

**Figure 2:**
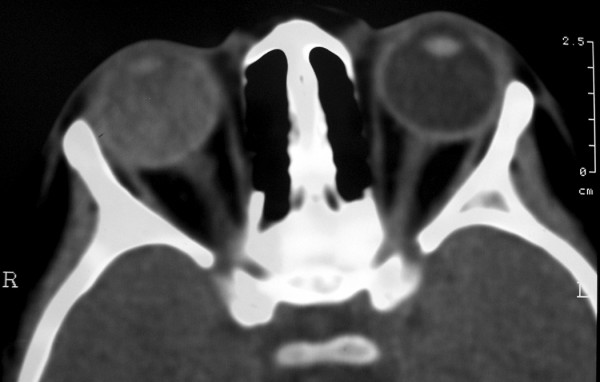
Computed Tomography. Total exsudative retinal detachment in the right eye.

A 4 year-old girl presented with a red and painful right eye. Visual acuity was no light perception in the right eye and 20/20 in the left. Slit lamp examination of the right eye revealed mild corneal edema, neovascular glaucoma, cataract and posterior synechiae (Fig. 1). Fundoscopy was impossible because of media opacity. Intraocular pressure was 35 mmHg in the right eye and 14 mmHg in the left one. Ocular examination of the left eye was unremarkable. Computerized Tomography showed total retinal detachment and heterogeneous subretinal fluid (Fig. 2). The eye was enucleated and a porous polyethylene orbital sphere was implanted.

**Figure 3:**
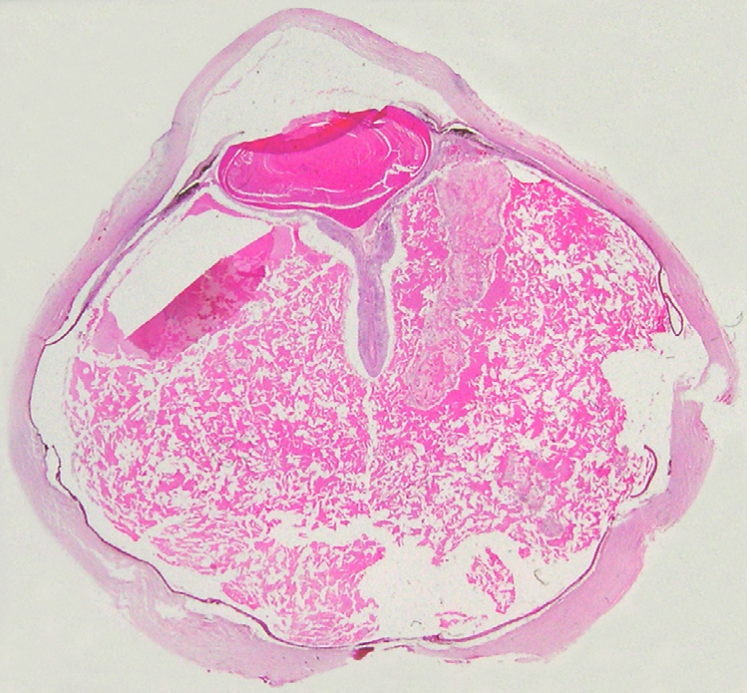
Coats' disease: histopathological findings. Total exsudative retinal detachment (H&E).

**Figure 4:**
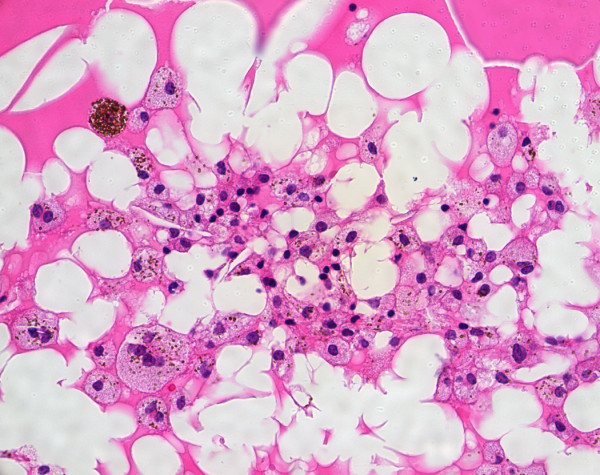
Coats' disease: histopathological findings. Subretinal fluid with cholesterol clefts and lipid-laden macrophages (H&E. Original magnification × 400).

**Figure 5:**
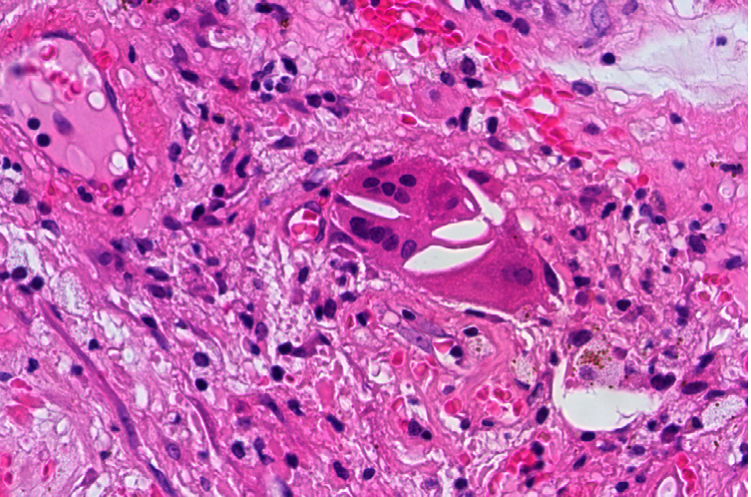
Coats' disease: histopathological findings. Intraretinal cholesterol deposition triggering a giant cell reaction foreign-body type (H&E. Original magnification × 400).

**Figure 6:**
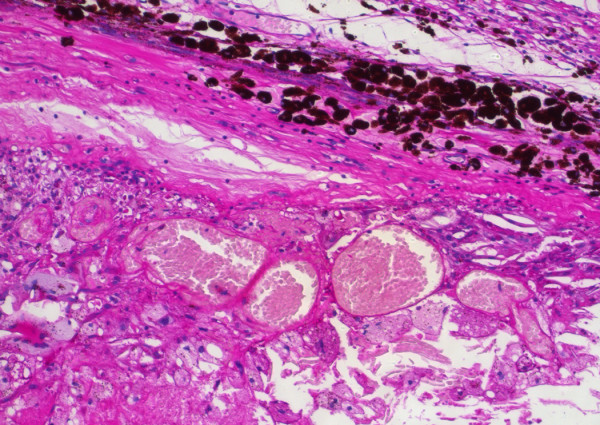
Coats' disease: histopathological findings. Telangiectasic retinal vessels (PAS. Original magnification × 200).

Histopathological evaluation disclosed total exudative retinal detachment and disorganization of the anterior segment (Fig. 3). The subretinal fluid was composed of PAS-positive material, cholesterol clefts and lipid-laden macrophages (Fig. 4). Lipid deposition was also seen in the retina inducing granulomatous inflammation foreign-body type (Fig. 5). In addition to the other findings, the presence of telangiectasic retinal vessels (Fig. 6) confirmed the diagnosis of Coats' disease.

Immunohistochemical studies were also performed. Vimentin was positive in all layers of the detached retina while Fibronectin expression was limited to the in the internal limiting membrane. The lipid-laden macropages in the subretinal fluid and within the retina were positive for CD-68. The telangiectasic vessels of the retina lacked the expression of Factor VIII.

## Conclusion

The exsudative retinal detachment in Coats' disease is caused by leakage of lipoproteic fluid from telangiectasic retinal vessels. These fusiform or saccular venous dilations tend to involve the temporal parafoveal quadrant of the retina and are especially common superotemporally. The presence of those vessel abnormalities differentiates Coats' disease from other causes of retinal detachment [2]. Immunohistochemical characteristics of the disease include the positive reaction for vimentin, fibronectin and CD68[5], which were verified by the results from the present study. A decrease in the number of endothelial cells in the telangiectasic retinal vessels was evident using Factor VII immunostaining. This finding supports previous studies using electron microscopy [6].

Clinically, the list of differential diagnosis of Coat's disease includes retinoblastoma, persistent hyperplastic primary vitreous (PHPV) and toxocariasis. Those diseases can be distinguished based on histopathological findings. Retinoblastoma is the most common primary intraocular malignancy of childhood [7]. Histologically, it is characterized by the presence of cells with round nuclei arranged in cuffs that surrounds retinal vessels [8]. Fleurettes, Flexner-Wintersteiner and Homer-Wright rosettes can be present depending on the degree of differentiation of the tumor [9]. PHPV, first described in 1955, consists of a congenital malformation of the primary vitreous that is characterized by a retrolental white plaque of fibrovascular tissue, visible through the pupil [10]. In cases with anterior PHPV, the ciliary processes are drawn inward by their attachment to the fibrotic tissue, often lying against the posterior lens, while, in cases with posterior PHPV, strands of glial tissue extend from the retina into the vitreous [11]. Toxocariasis is an infection by the nematode larvae of *Toxocara canis*. One of the manifestations of the ocular infection is a formation of a retrolental mass that constitutes the chronically inflamed and contracted vitreous, and an abcess or granuloma where the larva can be found [12].

General pathologists usually do not have the opportunity to receive and study specimens from patients with Coats' disease. Even ophthalmic pathologists rarely receive an enucleated eye because of Coats' disease. Ophthalmologists usually diagnose the disease in its earlier stages, enabling them to save the globe and even useful vision in most of the cases [13].

The diagnosis of Coats' disease and the exclusion of unilateral retinoblastoma in this particular case was made on clinical grounds and confirmed by histopathological evaluation. Although there are more conservative treatments to Coats' disease, enucleation is still indicated in cases with extensive exudative retinal detachment and secondary neovascular glaucoma [14]. The patient herein described presented as stage 4 of Coat's disease where treatments modalities other than enucleation were not effective.

Coats' disease is the second most common cause of pseudoretinoblastoma [15], being responsible for approximately 7% of the enucleations where the clinical diagnosis was retinoblastoma [16]. Thus, It is crucial for the pathologist to be familiar with the histopathological features of Coats' disease in order to differentiate it from retinoblastoma. The prognosis differs considerably from one disease to the other. Coats' disease has no associations with any systemic abnormalities. The patient is considered cured once the ocular manifestations are controlled and systemic treatment is unnecessary [4]. A misdiagnosis of retinoblastoma can submit a child to the potential risks and side effects of chemotherapy. On the other hand, retinoblastoma is a malignancy with a high mortality rate when not properly diagnosed and treated [17].

In summary, our report aims to document and illustrate the histopathological features of Coats' disease in a four year-old girl and to highlight the importance of establishing the correct differential diagnosis.

## Competing interests

The author(s) declare that they have no competing interests.

## Authors' contributions

BFF was responsible for the acquistion of clinical data and writing of the manuscript. ANO did the histopathological evaluation and drafting of the manunscript. SM assisted in the histopathological evaluation and revision of the manuscript. MZ participated in the design of the study and performed the critical revision of the manuscript. AGL assisted in the aquistion of clinical data and helped to draft the manuscript. MNBJr. participated in its design and coordination and helped to revise the manuscript. All authors read and approved the final manuscript.
